# Numerical simulations of the current-matching effect and operation mechanisms on the performance of InGaN/Si tandem cells

**DOI:** 10.1186/1556-276X-9-652

**Published:** 2014-12-02

**Authors:** Shih-Wei Feng, Chih-Ming Lai, Chin-Yi Tsai, Li-Wei Tu

**Affiliations:** 1 Department of Applied Physics, National University of Kaohsiung, Kaohsiung, Taiwan; 2 Department of Electronic Engineering, Ming Chuan University, Taoyuan, Taiwan; 3 Department of Physics and Center for Nanoscience and Nanotechnology, National Sun Yat-Sen University, Kaohsiung, Taiwan

**Keywords:** InGaN/Si tandem cell, III-V solar cell, Numerical simulation, Current-matching effect

## Abstract

Numerical simulations are conducted to study the current-matching effect and operation mechanisms in and to design the optimized device structure of InGaN/Si tandem cells. The characteristics of short circuit current density (*J*_sc_), open circuit voltage (*V*_oc_), fill factor (FF), and conversion efficiency (*η*) of InGaN/Si tandem cells are determined by the current-matching effect. The similar trend of *η* to that of *J*_sc_ shows that *J*_sc_ is a dominant factor in determining the performance of InGaN/Si tandem cells. In addition, the combined effects of the *J*_sc_, *V*_oc_, and FF lead to an optimized *η* in the medium-indium, $x_{p\left(n\right)-\text{InGaN}}^{\text{InGaN}‒to‒Si}$, InGaN/Si tandem cell. At $x_{p\left(n\right)-\text{InGaN}}^{\text{InGaN}‒to‒Si}$, the *J*_sc_ of the InGaN subcell is equal to that of the Si subcell such that an InGaN/Si tandem cell reaches the current matching condition to operate at the maximum power point. Similar to the *J*_sc_ and FF, the *η* for low- $\left(x_{p\left(n\right)-\text{InGaN}}<x_{p\left(n\right)‒\text{InGaN}}^{\text{InGaN}‒to‒Si}\right)$ and high-In $\left(x_{p\left(n\right)-\text{InGaN}}>x_{p\left(n\right)-\text{InGaN}}^{\text{InGaN}‒to‒Si}\right)$ InGaN/Si tandem cells are InGaN- and Si subcell-limited, respectively. Furthermore, the *p-* and *n*-layer thicknesses, indium content, and position of depletion region of InGaN subcell should be adjusted to reapportion the light between the two subcells and to achieve the maximum conversion efficiency. With appropriate thicknesses of *p*- and *n*-InGaN, In_0.5–0.6_Ga_0.5–0.4_ N/Si tandem cells can exhibit as high as approximately 34% to 36.5% conversion efficiency, demonstrating that a medium-indium InGaN/Si tandem cell results in a high-efficiency solar cell. Simulation results determine that the current-matching effect and operation mechanisms of InGaN/Si tandem cells can be utilized for efficiency enhancement through the optimized device structures.

## Background

The bandgap of InGaN semiconductors, ranging from 0.7 to 3.4 eV, can fit the full solar spectrum [1]. This provides InGaN semiconductors with a great advantage for photovoltaic applications. The development of InGaN solar cells is in the beginning stage. Our previous simulation results show that the performance and conversion efficiency of InGaN *p-i-n* homojunction solar cell strongly depend on the indium content, thickness, and defect density of the *i*-layer [2]. Also, our simulation results show that the performance and conversion efficiency of InGaN *p-n* junction solar cell is determined by the upper *p*-InGaN junction rather than the *n*-InGaN substrate [3]. An In_0.6_Ga_0.4_N *p-n* junction solar cell, with optimal device parameters, can have a conversion efficiency approximately 21.5%, demonstrating that medium-indium content InGaN materials have the potential to realize high-efficiency solar cells.

Device fabrications of *p*-*i*-*n* heterojunction, *p*-*i*-*n* homojunction, and *p*-*n* homojunction InGaN solar cells have been demonstrated [4-9]. Those reported that InGaN solar cells show a low conversion efficiency of less than 2%. Also, the reported conversion efficiency of Ga_0.83_In_0.17_ N (3 nm)/Ga_0.93_In_0.07_ N (1 nm) and Ga_0.83_In_0.17_ N (3 nm)/GaN (3 nm) superlattice solar cells is approximately 2.46% [10]. In addition, because of the lack of native substrates, the III-Nitride epilayers grown on sapphire substrates contain high densities of threading dislocation, stacking fault, and V-shaped defect, degrading the device performance [11,12]. Also, the low miscibility of InN and GaN leads to indium aggregation and phase separation, making it difficult to grow good quality high-indium InGaN [11,12]. Therefore, InGaN solar cells do not show as high a conversion efficiency as other conventional III-V solar cells and are usually low-indium content [4-9].

Since the solar spectrum (0 to 4 eV) is broad, a single junction solar cell cannot cover the whole solar spectrum. A tandem cell divides the solar spectrum into spectral ranges, each being converted in a different subcell, to achieve a high overall conversion efficiency. For an *m* series-connected multijunction solar cell, the voltage at a given current is equal to the sum of the subcell voltages at that current. The current through each of the subcells is constrained to have the same value. This is the current-matching condition [13]. Therefore, each subcell will be able to operate at its maximum power point and the maximum power output of the multijunction device is the sum of the maximum power outputs of the subcells. On the other hand, if the currents through each of the subcells do not all have the same value, the subcells cannot reach the current-matching condition and operate at their maximum power points.

Currently, the main problem in the conventional III-V tandem cells is the current-mismatching between subcells, which reduces significantly the conversion efficiency [14]. The performance of GaInP/GaAs two-junction series-connected cells has been well studied theoretically [13]. With the optimal bandgap combination of *E*_*g*_(GaInP) = 1.95 eV and *E*_*g*_(GaAs) = 1.42 eV, a 38% conversion efficiency is predicted, well in excess of the 29% efficiency for the best single-junction device. The current-matching condition plays an important role in determining the performance of GaInP/GaAs two-junction series-connected cells. This is due to the dependence of the top- and bottom-subcell photocurrents on the subcell bandgap and thickness. The short circuit current for the two-junction series-connected cell is the lesser of the top- and bottom-subcell-limited photocurrents.

Although numerical simulations of InGaN/InGaN two-junction solar cells, InGaN/Si tandem cells, and InGaN multiple-junction solar cells have been conducted [15-17], the current-matching effect of InGaN/Si tandem cells has not been well studied. To produce a high-efficiency InGaN/Si tandem cell, the current-matching effect and the operation mechanisms of InGaN/Si tandem cell must be well understood.

In this study, numerical simulations are conducted to determine the current matching-effect and operation mechanisms in and to design the optimized device structure in InGaN/Si tandem cells. The characteristics of short circuit current density (*J*_sc_), open circuit voltage (*V*_oc_), fill factor (FF), and conversion efficiency (*η*) of InGaN/Si tandem cells are determined by the current-matching effect, which in turn is affected by the *p-* and *n*-layer thicknesses, indium content, and position of depletion region of the InGaN subcell. *J*_sc_ is a dominant factor in determining the performance of InGaN/Si tandem cells. With appropriate thicknesses of *p*- and *n*-InGaN, 34.0% to 36.5% conversion efficiency of the In_0.5–0.6_Ga_0.5–0.4_ N/Si tandem cells suggests that medium-indium InGaN/Si is an appealing candidate to realize a high-efficiency solar cell.

This paper is organized as follows. In the ‘Methods’ section, theoretical modeling is described. In the ‘Results and discussion’ section, simulation results of the performance of InGaN/Si tandem cells are discussed. Finally, conclusions are drawn in the ‘Conclusions’ section.

## Methods

### Theoretical modeling of short circuit current density, open circuit voltage, fill factor, and conversion efficiency of InGaN/Si tandem cells

**Figure 1:**
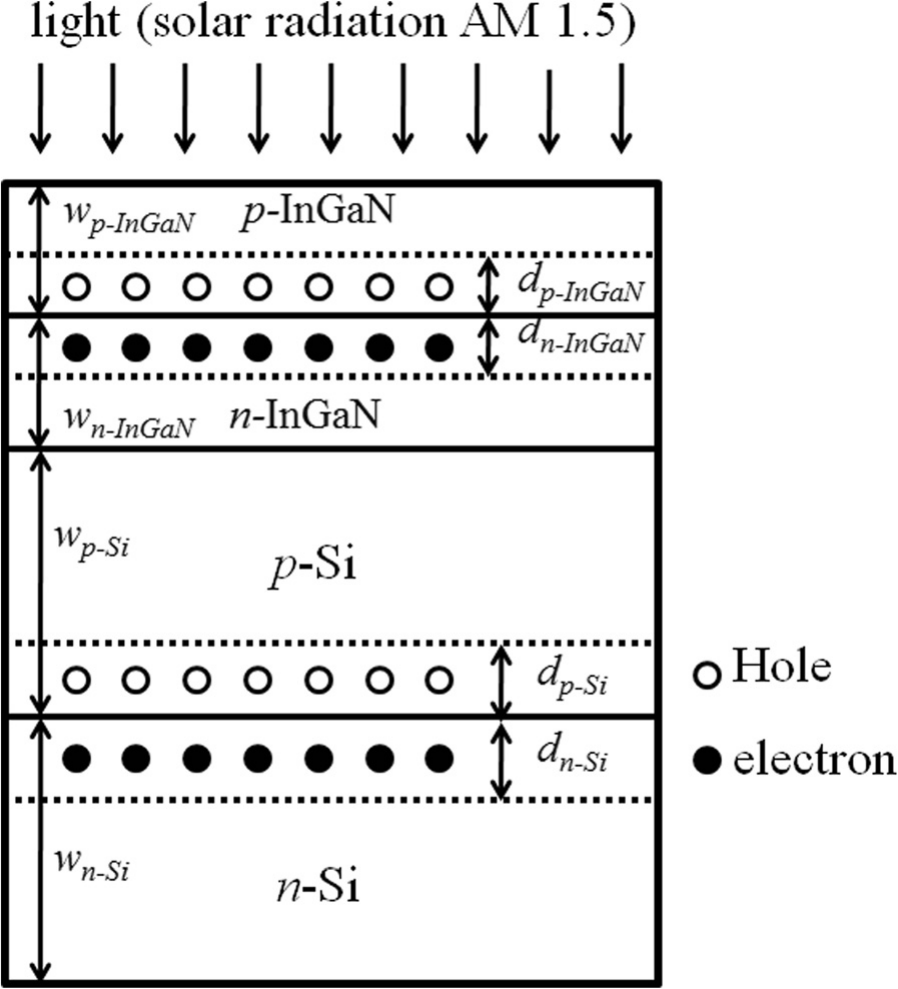
**The structure of InGaN/Si tandem cells used for theoretical simulation.** Light (AM 1.5G illumination) is incident from the *p*-InGaN side. *w*_*p-*InGaN(-Si)_ and *w*_*n-*InGaN(-Si)_ are the thicknesses of the *p*- and *n*-InGaN(-Si) junctions, respectively. *d*_*p-*InGaN(-Si)_ and *d*_*n-*InGaN(-Si)_ are the thicknesses of the depletion region in the *p*- and *n*-InGaN(-Si) junctions, respectively.

Figure 1 shows the structure of InGaN/Si tandem cells used for the theoretical simulation. *w*_*p-*InGaN(*-*Si)_ and *w*_*n-*InGaN(*-*Si)_ are the thicknesses of the *p*- and *n*-InGaN(-Si) junctions, respectively. *d*_*p-*InGaN(*-*Si)_ and *d*_*n-*InGaN(*-*Si)_ are the thicknesses of the depletion region in the *p*- and *n*-InGaN(-Si) junctions, respectively. The solar cells are under solar radiation AM 1.5G illumination (100 mW/cm^2^). Photons are assumed to be incident from the *p*-InGaN side of the InGaN top cell.

In the numerical simulations, the theoretical model is used to design the structures of *p-*InGaN/*n-*InGaN/*p-*Si/*n-Si* tandem cells. Similar to the InGaN *p*-*n* junction solar cell in our previous study, the first-principles continuity and Poisson’s equations are combined to analyze the transport behavior of the InGaN top cell and Si bottom cell [3,18]. The photovoltaic functions of the InGaN and Si subcells can be analyzed by solving a set of coupled differential equations for the electron density, hole density, and electrostatic potential [3,18]. Carrier and current densities can be analytically obtained to separately determine the current-voltage (*J*-*V*) curves of the InGaN top cell and Si bottom cell.

The current density, *J*_InGaN_, in the InGaN *p-n* junction solar cells can be expressed as [3,18]:

(1) $$\begin{array}{ll} J_{\text{InGaN}} & =J_{SCP}+J_{SCN}+J_{G,D}\;-\;\left(J_{DP}+J_{DN}\right)\\ & \;\times \left(e^{qV_{a}/kT}-1\right)\;-\;J_{DD}\left(e^{qV_{a}/2kT}-1\right) \end{array}$$

where *J*_SCP_ is the hole diffusion current density in the *p*-InGaN junction, *J*_SCN_ is the electron diffusion current density in the *n*-InGaN junction, and *J*_G,D_ is the drift current density in the depletion region. *J*_DP_, *J*_DN_*,* and *J*_DD_ are the dark current densities in the *p*-InGaN junction, *n*-InGaN junction, and depletion region, respectively. *V*_*a*_ is the built-in potential. Each term of *J*_SCP_, *J*_SCN_, *J*_G,D_, *J*_DP_, *J*_DN_, and *J*_DD_ in Equation 1 can be obtained in references [3] and [18]. From Equation 1, *J*_InGaN_ can be expressed as:

(2) $$J_{\text{InGaN}}=J_{sc}-\;J_{s1}\left(e^{qV_{a}/kT}-1\right)\;-\;\;J_{s2}\left(e^{qV_{a}/2kT}-1\right)$$

(3) $$J_{sc}\equiv J_{SCP}+J_{SCN}+J_{G,D}$$

(4) $$J_{s1}\equiv J_{DP}+J_{DN}$$

(5) $$J_{s2}\equiv J_{DD}$$

where *J*_SC_ is the photocurrent, $J_{s1}\left(e^{qV_{a}/kT}-1\right)$ is the dark current in the neutral region, and $J_{s2}\left(e^{qV_{a}/2kT}-1\right)$ is the recombination current in the depletion region. Details of the calculations of total current density, *J*_InGaN_, were described in references [3] and [18].

Similar to the InGaN *p*-*n* junction top cell, the first-principles continuity and Poisson’s equations are combined to analyze the transport behavior of the Si bottom cell [3,18]. The current density, *J*_Si_, in the Si *p-n* junction solar cells can be obtained [3,18].

The InGaN/Si tandem cell must consider the current-matching effect. The short circuit current density, *J*_sc_, of an InGaN/Si tandem cell is limited by the smaller short circuit current density in the InGaN and Si subcells. It should be noted that the series and shunt resistances of the devices are not included in the following discussion in order to focus on the effects of the ideal diode characteristics of devices. However, it should be reminded that in the cases of thick InGaN layers or poor metal contacts resulted from the *p*-doing InGaN, the effect of series resistances will become significant and their effects thus should be fully taken into account. In addition, the important yet complicate issue regarding the tunnel junction is not discussed in this work; therefore, the tunnel junction between the InGaN and Si is assumed to be an ideal one which has no additional effect on the devices’ performance. Of course, such an assumption is oversimplified. Since the electron affinity of the InGaN varies widely with the indium composition, it is very difficult to achieve good tunnel junctions between the InGaN and Si and their effects on the devices’ performance will be significant and thus deserve a separate and dedicate work to discuss this issue.

Assuming that the recombination current in the depletion region $\left(J_{s2}\left(e^{qV_{a}/2kT}-1\right)≅0\right)$ is very small, the open-circuit voltage, *V*_oc_, can be obtained by setting the *J*_InGaN_ in Equation 2 to be zero [3,18].

(6) $$\begin{array}{ll} J_{\text{InGaN}}=J_{sc}\; & -\;J_{s1}\left(e^{qV_{a}/kT}-1\right)\;-\;J_{s2}\left(e^{qV_{a}/2kT}-1\right)\;\approx \;J_{sc}\;-\;J_{s1}\\ & \;\left(e^{qV_{a}/kT}-1\right)\equiv 0 \end{array}$$

(7) $$\Rightarrow V_{oc}=\frac{kT}{q}\ln\;\frac{J_{sc}+J_{s1}}{J_{s1}}$$

when *J*_sc_ > > *J*_s1_

(8) $$\Rightarrow V_{oc}≅\frac{kT}{q}\ln\;\frac{J_{sc}}{J_{s1}}$$

The *V*_oc_ of InGaN/Si tandem cell is the sum of the *V*_oc_*s* of InGaN and Si subcells [3,18].

The fill factor, FF, is defined as:

(9) $$FF=\frac{P_{\text{max}}}{V_{oc}\cdot I_{sc}}=\frac{V_{\text{max}}\cdot I_{\text{max}}}{V_{oc}\cdot I_{sc}}=\frac{V_{\text{max}}\cdot J_{\text{max}}}{V_{oc}\cdot J_{sc}}$$

The power conversion efficiency of a solar cell, *η*, is defined as [2,3]:

(10) $$\eta =\frac{P_{\text{max}}}{P_{\mathrm{in}}}=\frac{FF\cdot V_{oc}\cdot I_{sc}}{P_{\mathrm{in}}}$$

The intrinsic carrier concentration, *n*_*i*_, can be described by [2,3]:

(11) $$n_{i}^{2}=2.31\times 10^{31}{\left(\frac{m_{n}\cdot m_{p}}{m_{e}^{2}}\right)}^{\;2/3}\times T^{3}\times \exp\left(-\;\frac{E_{g}}{kT}\right)$$

The bandgap energy, *E*_*g*_*(x)*, for In_x_Ga_1-x_N is expressed as [1]:

(12) $$E_{g}\left(x\right)=0.65x+3.425\left(1\;-\;x\right)\;-\;1.43x\left(1\;-\;x\right)$$

The absorption coefficients *α*(*E*) for direct InGaN and indirect Si as a function of energy, *E*, can be expressed as Equations 13 and 14, respectively,

(13) $$\alpha \left(E\right)=\alpha_{0}\sqrt{\frac{E\;-\;E_{g}\left(x\right)}{E_{g}\left(x\right)}}$$

(14) $$\alpha \left(E\right)=\alpha_{0}{\left(\frac{E\;-\;E_{g}\left(x\right)}{E_{g}\left(x\right)}\right)}^{2}$$

where *E*_*g*_(*x*) are the bandgaps of In_*x*_Ga_1-*x*_N and Si [1]. The constant factor *α*_0_ is shown in Table 1. Except for the bandgap energy, the physical parameters of In_*x*_Ga_1-*x*_N are expressed as the linear interpolation formula of InN and GaN. The physical parameters of InN, GaN, and Si are listed in Table 1[1,19-24]. The *p*- and *n*-Si thicknesses are set at 0.7 and 200 μm, respectively.

**Table 1:** The parameters of InN and GaN used for theoretical simulations

	**InN**	**GaN**	**Si**
Electron effective mass *m*_*n*_	0.11 m_e_[1]	0.2 m_e_[1]	0.98m_e_[1]
Hole effective mass *m*_*p*_	1.63 m_e_[1]	0.8 m_e_[1]	0.49m_e_[1]
Dielectric constant *E*_*si*_	15.3 [1]	8.9 [1]	11.9 [1]
Hole lifetime *τ*_*p*_ (ns)	5.4 [17]	2 [18]	10^3^[1]
Electron lifetime *τ*_*n*_ (ns)	1.3 [19]	0.1 [20]	10^3^[1]
Hole diffusion constant *D*_*p*_ (cm^2^ · s^-1^)	8 [21]	0.75 [1]	12 [1]
Electron diffusion constant *D*_*n*_ (cm^2^ · s^-1^)	80 [21]	39 [4]	39 [1]
Hole surface recombination rate *S*_*p*_ (cm · s^-1^)	10^3^	10^3^	10^3^
Electron surface recombination rate *S*_*n*_ (cm · s^-1^)	10^3^	10^3^	10^3^
Donor concentration *N*_*D*_ (cm^-3^)	5 × 10^17^[19]	5 × 10^17^[19]	6 × 10^17^[19]
Acceptor concentration *N*_*A*_ (cm^-3^)	5 × 10^17^[19]	5 × 10^17^[19]	6 × 10^17^[19]
Absorption coefficient *α*_0_ (cm^-1^)	1.5 × 10^5^[1]	2 × 10^5^[1]	1 × 10^3^[1]

Operation mechanisms of InGaN *p-n* junction solar cells are explored through the calculation of characteristic parameters such as the *J*_sc_, *V*_oc_, FF, and *η*. Two situations are considered for theoretical simulation:

(I) Situation I: the dependence on the thickness (*w*_*p-*InGaN_ = 50 to 4,000 nm) and the indium composition (*x*_*p-*InGaN_ = 0, 0.1….0.9, 1) of the *p-*InGaN junction. The *n*-InGaN thickness is set at 1,000 nm. The various thicknesses of the *p-*InGaN junction keep the depletion region of InGaN junction at the same distance from the Si bottom subcell while changing the amount of light absorbed close to the depletion region of InGaN junction.

(II) Situation II: the dependence on the thicknesses (*w*_*n-*InGaN_ = 50 to 4,000 nm) and the indium composition (*x*_*n-*InGaN_ = 0, 0.1….0.9, 1) of the *n-*InGaN junction. The *p*-InGaN thickness is set at 300 nm. The various thicknesses of *n-*InGaN junction move the depletion region of InGaN junction relative to the Si bottom subcell while keeping the amount of light absorbed close to the depletion region of InGaN junction constant.

## Results and discussion

### (I) The effects of the thickness and the indium composition of the *p-*InGaN junction on the performance of InGaN/Si tandem cells

**Figure 2:**
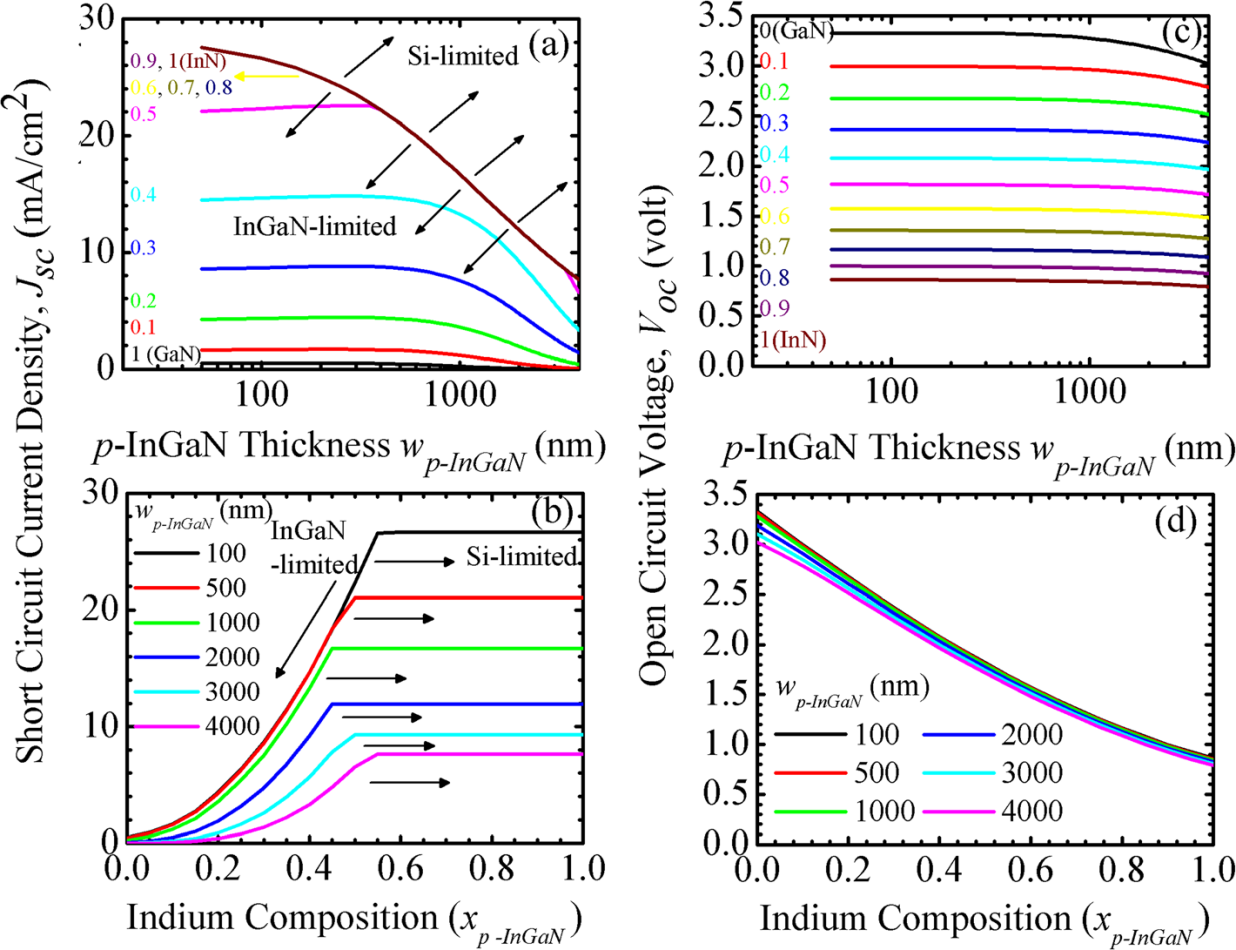
**Short circuit current density.***J*_sc_(*w*_*p-*InGaN_, *x*_*p*-InGaN_) of InGaN/Si tandem cells as a function of *p*-InGaN **(a)** thickness *w*_*p-*InGaN_ and **(b)** indium composition *x*_*p-*InGaN_. Open circuit voltage, *V*_oc_(*w*_*p-*InGaN_, *x*_*p-*InGaN_), as a function of **(c)***w*_*p*-InGaN_ and **(d)***x*_*p-*InGaN_.

First, simulation I is conducted. Figure 2a shows the short circuit current density, *J*_sc_(*w*_*p-*InGaN_, *x*_*p-*InGaN_), of InGaN/Si tandem cells as a function of *p*-InGaN thickness (*w*_*p-*InGaN_). The current-matching effect determines the behavior of the *J*_sc_. The discussions are divided into low- (*x*_*p-*InGaN_ = 0 to 0.4), medium- (*x*_*p-*InGaN_ = 0.5), and high-indium (*x*_*p-*InGaN_ = 0.6 to 1.0) InGaN category regions:

(I) For *x*_*p-*InGaN_ = 0 to 0.4, the *J*_sc_ very slightly increases and then drops with increasing *w*_*p-*InGaN_. The higher bandgap of the low-In InGaN results in the light passing through it being less absorbed. The *J*_sc_ of InGaN/Si tandem cells is the smaller of the InGaN subcell-limited photocurrents. Due to the photons being incident from the *p*-InGaN of the solar cell, photogenerated carriers in the *p*-InGaN contribute more photocurrent than those in the *n*-InGaN. As *w*_*p-*InGaN_ increases, a thicker *w*_*p-*InGaN_ increases absorption. The *J*_sc_ very slightly increases. In addition, with a further increase in *w*_*p-*InGaN_ (>1,000 nm), the depletion region of InGaN subcell is further away from the top cell surface. The collection efficiency of the minority carriers in the depletion region of InGaN subcell decreases and the probability of carrier recombination at surface defects increases. Thus, the *J*_sc_ decreases.

(II) For *x*_*p-*InGaN_ = 0.5, as the *w*_*p-*InGaN_ increases, the *J*_sc_ slightly increases and then drops. With smaller (<400 nm), medium-sized (400 nm to 3 μm), and larger (>3 μm) *w*_*p-*InGaN_, the *J*_sc_ of an In_0.5_Ga_0.5_N/Si tandem cell is shown to be InGaN-, Si-, and InGaN subcell-limited, respectively. Two turning points, at current-matching condition around 400 nm and 3 μm, are observed. (i) For *w*_*p-*InGaN_ < 400 nm, because the absorption coefficient *α(hν)* for solar cell materials is finite, a cell of finite thickness will not absorb all the incident light above the bandgap. A thicker InGaN subcell increases the absorption and the *J*_sc_ slightly increases. Due to the medium bandgap, In_0.5_Ga_0.5_N (*E*_*g*_ = 1.68 eV) may not absorb so many photons such that the *J*_sc_ in the InGaN subcell is smaller than that in the Si subcell. The *J*_sc_ is InGaN-subcell-limited. (ii) With a further increase in *w*_*p-*InGaN_ to (>400 nm), the lower transmission to the Si bottom subcell leads to the photocurrent in the Si subcell being lower than that in the InGaN subcell, so that the *J*_sc_ becomes Si-subcell-limited. (iii) For *w*_*p-*InGaN_ >3 μm, the depletion region of InGaN subcell is further away from the top cell surface. The collection efficiency of the minority carriers in the depletion region of InGaN subcell decreases, and the probability of carrier recombination at surface defects increases. Hence, the *J*_sc_ decreases and is again InGaN subcell-limited. Therefore, for the In_0.5_Ga_0.5_N/Si tandem cell, the thickness of the *p*-InGaN junction (*w*_*p-*InGaN_) should be adjusted to reapportion the light between the two subcells and to achieve the maximum conversion efficiency.

(III) For *x*_*p-*InGaN_ = 0.6 to 1.0, the *J*_sc_s are all the same and decrease with increasing *w*_*p-*InGaN_. A thicker *w*_*p-*InGaN_ and the lower bandgap of the high-In InGaN top cell absorbs more light, so less light is transmitted to the Si bottom cell. The photocurrent generated from the Si subcell becomes smaller and the *J*_sc_ of the overall cell decreases further. The *J*_sc_s are Si subcell-limited.

Figure 2b shows the short circuit current density, *J*_sc_(*w*_*p-*InGaN_, *x*_*p-*InGaN_), of InGaN/Si tandem cells as a function of indium composition (*x*_*p-*InGaN_). For the same thickness of *p*-InGaN, as the *x*_*p-*InGaN_ increases, the *J*_sc_ increases and then decreases very slightly at a certain composition, $x_{p-\text{InGaN}}^{\text{InGaN}‒to‒Si}$. It should be noted that once the band-gap energy of the InGaN top subcell is smaller than that of the Si bottom subcell, the top cell will absorb a certain potion spectrum of the incident photons which originally only can be absorbed by the Si bottom subcell. As a result, the short circuit current densities decrease very slightly for higher In compositions after reaching the maximum point. The respective low and high absorptions of the low- $\left(x_{p-\text{InGaN}}<x_{p-\text{InGaN}}^{\text{InGaN}‒to‒Si}\right)$ and high-In $\left(x_{p-\text{InGaN}}>x_{p-\text{InGaN}}^{\text{InGaN}‒to‒Si}\right)$ InGaN lead to the *J*_sc_ being InGaN and Si subcell-limited, respectively. As the *w*_*p-*InGaN_ decreases, the $x_{p-\text{InGaN}}^{\text{InGaN}‒to‒Si}$ decreases and then increases. This is determined by the current-matching effect, as shown in Figure 2a.

Figure 2c,d shows the open circuit voltage, *V*_oc_(*w*_*p-*InGaN_, *x*_*p-*InGaN_), of InGaN/Si tandem cells as a function of *w*_*p-*InGaN_ and *x*_*p-*InGaN_, respectively. The *V*_oc_ of an InGaN/Si tandem cell is equal to the sum of the *V*_oc_s of the InGaN and Si subcells. In Figure 2c, except for the thicker cells, the *V*_oc_ of an InGaN/Si tandem cell is nearly independent of the *w*_*p-*InGaN_. Note that *V*_oc_ starts to decrease slightly in the thicker cell, due to the larger saturation current, *J*_0_, in the thicker cell. In Figure 2d, because *V*_oc_ is determined by the bandgap energy of the subcell [18], smaller *V*_oc_ in the high-In InGaN top cell is expected.

**Figure 3:**
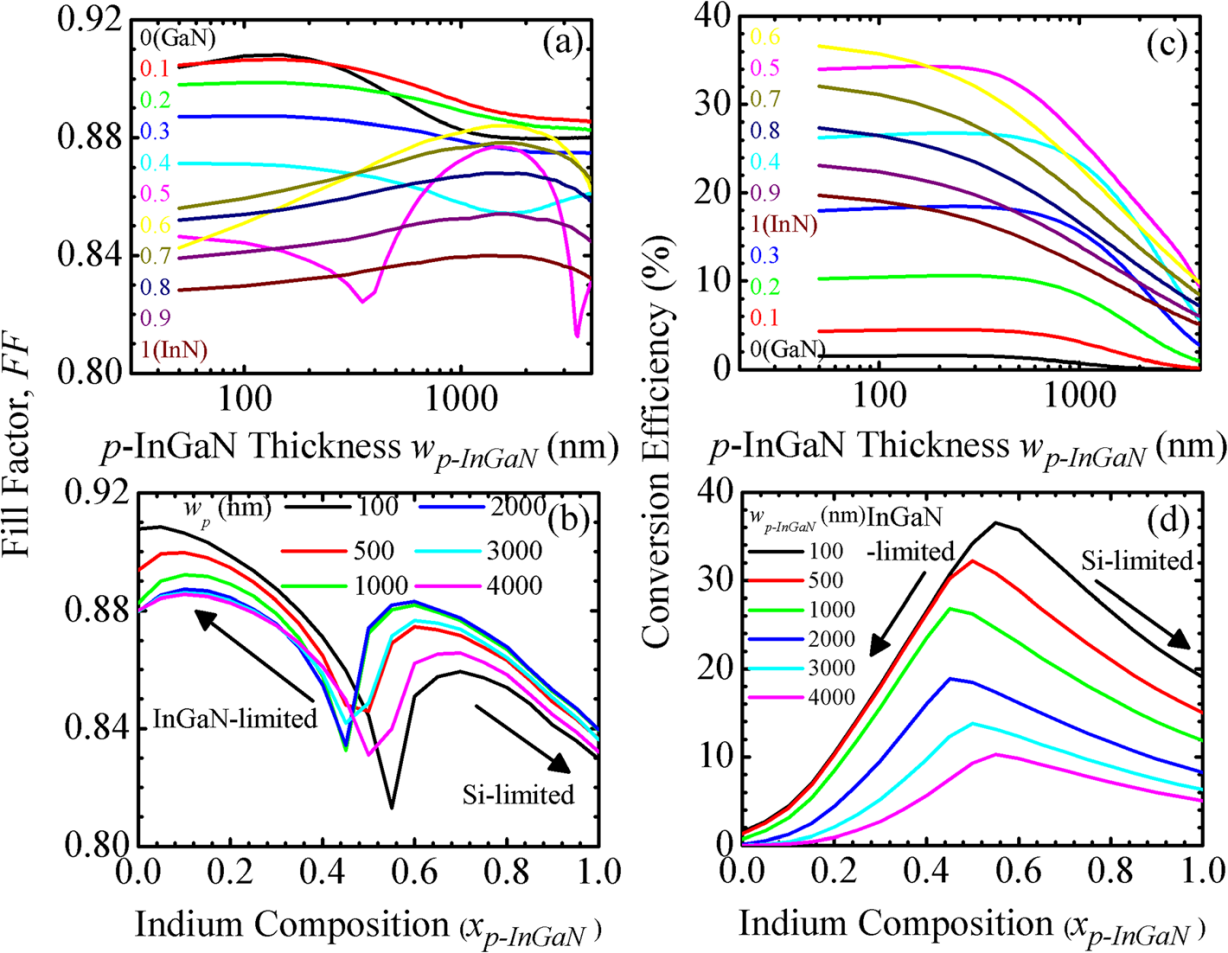
**Fill factor.** FF(*w*_*p-*InGaN_, *x*_*p-*InGaN_) of InGaN/Si tandem cells as a function of *p*-InGaN **(a)** thickness, *w*_*p-*InGaN_ and **(b)** indium composition, *x*_*p-*InGaN_. Conversion efficiency, *η*(*w*_*p-*InGaN_, *x*_*p-*InGaN_), as a function of **(c)***w*_*p-*InGaN_ and **(d)***x*_*p-*InGaN_.

Figure 3a,b shows the fill factor, FF(*w*_*p-*InGaN_, *x*_*p-*InGaN_), of InGaN/Si tandem cells as a function of *w*_*p-*InGaN_ and *x*_*p-*InGaN_, respectively. The behaviors of FF are dramatic. According to Equations 9 and 10, the FF represents the combined effects of *P*_max_, *J*_sc_, and *V*_oc_, which in turn are affected by the current-matching effect. Because the power maximum, *P*_max_, in Equation 10 is proportional to the conversion efficiency, *η*, the FF in Equation 9 represents the ratio of *η* to (*J*_sc_*V_oc_). As shown in Figure 2c, except for the thicker *w*_*p-*InGaN_, the *V*_oc_ of an InGaN/Si tandem cell is nearly independent of the *w*_*p-*InGaN_, the effect of *V*_oc_ can be neglected and the FF represents the ratio of *η* to *J*_sc_. In Figure 3a, the FFs are divided into low- (*x*_*p-*InGaN_ = 0 to 0.4), medium- (*x*_*p-*InGaN_ = 0.5), and high-indium (*x*_*p-*InGaN_ = 0.6 to 1.0) InGaN categories:

(I) For *x*_*p-*InGaN_ = 0 to 0.4, the FF are InGaN subcell-limited. For *x*_*p-*InGaN_ = 0, 0.1, and 0.2, as the *w*_*p-*InGaN_ increases, the FF slightly increases and then decreases. For *x*_*p-*InGaN_ = 0.3 and 0.4, a minimum FF around 1 to 2 μm *w*_*p-*InGaN_ is observed.

(II) For *x*_*p-*InGaN_ = 0.5, the FF shows a dramatic variation with two minimums at current-matching condition around 400 nm and 3 μm. With smaller (<400 nm), medium-sized (400 to 3,000 nm), and larger (>3 μm) *w*_*p-*InGaN_, the FF of an In_0.5_Ga_0.5_N/Si tandem cell is shown to be InGaN-, Si-, and InGaN subcell-limited, respectively. Because *J*_sc_ has two turning points around 400 nm and 3 μm, two minimum FF at current-matching condition are expected.

(III) For *x*_*p-*InGaN_ = 0.6 to 1.0, the FF are Si subcell-limited. As the *w*_*p-*InGaN_ increases, the FF increases and then decreases at 1 to 2 μm.

In Figure 3b, the FFs for low- $\left(x_{p-\text{InGaN}}<x_{p-\text{InGaN}}^{\text{InGaN}‒to‒Si}\right)$ and high-In $\left(x_{p-\text{InGaN}}>x_{p-\text{InGaN}}^{\text{InGaN}‒to‒Si}\right)$ InGaN/Si tandem cells are InGaN and Si subcell-limited, respectively. Minimum FF at the current-matching condition is observed at a certain composition, $x_{p-\text{InGaN}}^{\text{InGaN}‒to‒Si}$. The $x_{p-\text{InGaN}}^{\text{InGaN}‒to‒Si}$ is also determined by the same $x_{p-\text{InGaN}}^{\text{InGaN}‒to‒Si}$, as shown in Figure 2b. In general, the III-V solar cells exhibit a high FF of 0.80 to 0.86 [25]. Without consideration of the effects of the current leakage and shunt resistance, the simulation results of FF can be higher than those of the actual fabricated solar cells.

Figure 3c,d shows the conversion efficiency, *η*(*w*_*p-*InGaN_, *x*_*p-*InGaN_), of an InGaN/Si tandem cell as a function of *w*_*p-*InGaN_ and *x*_*p-*InGaN_, respectively. Simulation results help us to better understand the current-matching effect and operation mechanisms in and provide the optimized structure design of InGaN/Si tandem cells. The *η* represents the combined effects of *J*_sc_, *V*_oc_, and FF, which in turn are affected by the current-matching effect.

In Figure 3c, the trend of *η* is similar to that of *J*_sc_ in Figure 2a. This shows that *J*_sc_ is a dominant factor in determining the performance of InGaN/Si tandem cells. The *η* is divided into low- (*x*_*p-*InGaN_ = 0 to 0.4), medium- (*x*_*p-*InGaN_ = 0.5), and high-indium (*x*_*p-*InGaN_ = 0.6 to 1.0) InGaN categories:

(I) For *x*_*p-*InGaN_ = 0 to 0.4, the InGaN subcell-limited *η* slightly increases and then drops with increasing *w*_*p-*InGaN_. Due to very lower *η*, low-indium content (*x*_*p-*InGaN_ = 0 to 0.3) InGaN/Si tandem cells are not suitable for application in solar cells.

(II) For *x*_*p-*InGaN_ = 0.5, as the *w*_*p-*InGaN_ increases, the *η* slightly increases and then drops. With smaller (<400 nm), medium-sized (400 to 3,000 nm), and larger (>3 μm) *w*_*p-*InGaN_, the *η* is shown to be InGaN-, Si-, and InGaN subcell-limited, respectively. Two turning points at current-matching condition around 400 nm and 3 μm are observed. The current-matching effect determines the behavior of the *η*. With 100 to 300 nm *p*-InGaN and 300 nm *n*-InGaN, the In_0.5_Ga_0.5_N(1.68 eV)/Si(1.12 eV) tandem cell can exhibit as high a *η* as approximately 34%.

For *x*_*p-*InGaN_ = 0.6 to 1, the Si subcell-limited *η* decreases with increasing *w*_*p-*InGaN_. With 50 nm *p*-InGaN and 300 nm *n*-InGaN, the In_0.6_Ga_0.4_N(1.42 eV)/Si(1.12 eV) tandem cell can exhibit as high a *η* as approximately 36.5%, which is much higher than the approximately 22% conversion efficiency of an In_0.6_Ga_0.4_N *p*-*n* single junction solar cell and comparable to the 35% to 38% conversion efficiency of a GaInP(1.95 eV)/GaAs(1.42 eV) tandem cell [3,13].

In Figure 3d, the combined effects of the *J*_sc_, *V*_oc_, and FF lead to an optimized *η* in the medium-indium, $x_{p-\text{InGaN}}^{\text{InGaN}‒to‒Si}$, InGaN/Si tandem cell. At $x_{p-\text{InGaN}}^{\text{InGaN}‒to‒Si}$, the *J*_sc_ of the InGaN subcell is equal to that of the Si subcell such that the InGaN/Si tandem cell reaches the current matching condition to operate at the maximum power point. Similar to *J*_sc_ and FF, the *η* for low- $\left(x_{p-\text{InGaN}}<x_{p-\text{InGaN}}^{\text{InGaN}‒to‒Si}\right)$ and high-In $\left(x_{p-\text{InGaN}}>x_{p-\text{InGaN}}^{\text{InGaN}‒to‒Si}\right)$ InGaN/Si tandem cells are InGaN and Si subcell-limited, respectively. The $x_{p-\text{InGaN}}^{\text{InGaN}‒to‒Si}$ in Figure 3d is the same as the $x_{p-\text{InGaN}}^{\text{InGaN}‒to‒Si}$ in Figure 2b. With 100 nm *p*-InGaN and 300 nm *n*-InGaN, the In_0.55_Ga_0.45_N(1.54 eV)/Si(1.12 eV) tandem cell can exhibit as high a *η* as approximately 36.5%, which is much higher than approximately 22% conversion efficiency of an In_0.6_Ga_0.4_N *p*-*n* single junction solar cell and comparable to the 35% to 38% conversion efficiency of GaInP(1.95 eV)/GaAs(1.42 eV) tandem cell [3,13]. This demonstrates that the medium-indium InGaN/Si tandem cell is an appealing candidate to realize a high-efficiency solar cell. However, the difficulty of high-quality devices would be a potential obstacle to fabricating such tandem cells. Growth of In-rich InGaN can be obtained by using high-pressure chemical vapor deposition [26,27].

### (II) The effects of the thickness and the indium composition of the *n-*InGaN junction on the performance of InGaN/Si tandem cells

**Figure 4:**
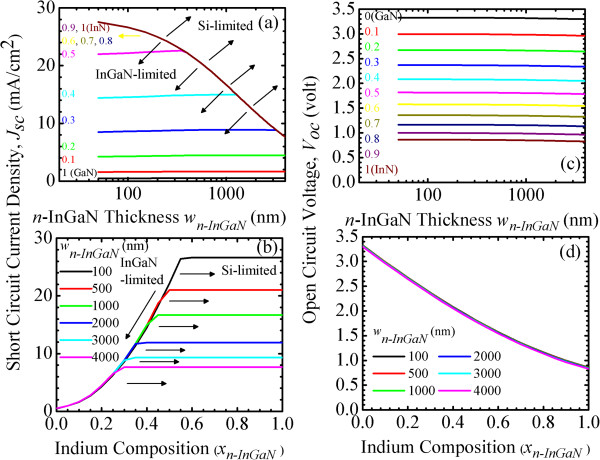
**Short circuit current density.***J*_sc_(*w*_*n-*InGaN_, *x*_*n-*InGaN_) of InGaN/Si tandem cells as a function of *n*-InGaN **(a)** thickness, *w*_*n-*InGaN_, and **(b)** indium composition, *x*_*n-*InGaN_. Open circuit voltage, *V*_oc_(*w*_*n-*InGaN_, *x*_*n-*InGaN_), as a function of **(c)***w*_*n-*InGaN_ and **(d)***x*_*n-*InGaN_.

Secondly, simulation II is conducted. Figure 4a,b shows the short circuit current densities, *J*_sc_(*w*_*n-*InGaN_, *x*_*n-*InGaN_), of InGaN/Si tandem cells as a function of *n*-InGaN thickness (*w*_*n-*InGaN_) and indium composition (*x*_*n-*InGaN_), respectively. In Figure 4a, current-matching effect categorizes the *J*_sc_ into InGaN and Si subcell-limited areas. The discussions are divided into low- (*x*_*n-*InGaN_ = 0 to 0.2), medium- (*x*_*n-*InGaN_ = 0.3 to 0.5), and high-indium (*x*_*p-*InGaN_ = 0.6 to 1) InGaN regions:

(I) For *x*_*n-*InGaN_ = 0 to 0.2, the *J*_sc_ slightly increases with increasing *w*_*n-*InGaN_, due to the increase of photon absorption by thicker *n*-InGaN layer. The higher bandgap of the low-In InGaN results in the light passing through it being less absorbed. The *J*_sc_ is InGaN subcell-limited.

(II) For *x*_*n-*InGaN_ = 0.3 to 0.5, as the *w*_*n-*InGaN_ increases, the *J*_sc_ slightly increases and then drops beyond a certain thickness $\left(w_{n-\text{InGaN}}^{\text{InGaN}‒to‒Si}\right)$. The current-matching effect categorizes the *J*_sc_ into InGaN and Si subcell-limited areas. A larger *w*_*n-*InGaN_ absorbs more photons such that the *J*_sc_ slightly increases. As the *w*_*n-*InGaN_ is beyond a certain thickness $\left(w_{n-\text{InGaN}}^{\text{InGaN}‒to‒Si}\right)$, the enhanced absorption of *n*-InGaN leads to less light being transmitted to the Si bottom subcell. The *J*_sc_ is Si subcell-limited. Also, the higher the *x*_*n-*InGaN_, the smaller the $\left(w_{n-\text{InGaN}}^{\text{InGaN}‒to‒Si}\right)$ can be. Due to the lower bandgap of the high-In InGaN, a smaller *w*_*n-*InGaN_ can absorb more photons to reach the current-matching condition.

(III) For *x*_*p-*InGaN_ = 0.6 to 1, the *J*_sc_s are all the same and decrease with increasing *w*_*n-*InGaN_. A thicker *w*_*n-*InGaN_, combined with the low bandgap of high-In InGaN, absorbs more photons and leads to less transmittance to the Si bottom subcell. The photocurrent generated from the Si subcell becomes smaller and the *J*_sc_ of the overall cell decreases further. The *J*_sc_ is Si subcell-limited.

In Figure 4b, as the *x*_*n-*InGaN_ increases, the *J*_sc_ increases and then *decreases very slightly* at a certain composition, $x_{n-\text{InGaN}}^{\text{InGaN}‒to‒Si}$, at which the *J*_sc_ ceases to increase. Similar to the *J*_sc_(*w*_*p-*InGaN_, *x*_*p-*InGaN_) in Figure 2b, the current-matching effect can also explain the trend of *J*_sc_(*w*_*n-*InGaN_, *x*_*n-*InGaN_). Also, the thinner the *w*_*n-*InGaN_, the higher the indium composition, $x_{n-\text{InGaN}}^{\text{InGaN}‒to‒Si}$, to reach the current matching condition. The thickness and bandgap of the *n*-InGaN layer can determine the light flux reaching the Si subcell. With a thinner *w*_*n-*InGaN_, more light is able to reach the Si subcell to generate more photocurrent. The *J*_sc_ is more easily InGaN subcell-limited in the low-indium InGaN/Si tandem cell. To make the *J*_sc_ become Si subcell-limited, a lower bandgap in the higher indium content InGaN subcell is needed to absorb more photons. Hence, for the thinner *w*_*n-*InGaN_, the *J*_sc_ transition from InGaN to Si subcell-limited is observed at a higher indium composition.

Figure 4c,d shows the open circuit voltage, *V*_oc_(*w*_*n-*InGaN_, *x*_*n-*InGaN_), of InGaN/Si tandem cells as a function of *w*_*n-*InGaN_ and *x*_*n-*InGaN_, respectively. The trend of *V*_oc_(*w*_*n-*InGaN_, *x*_*n-*InGaN_) is similar to that of *V*_oc_(*w*_*p-*InGaN_, *x*_*p-*InGaN_). A similar argument can explain this trend.

**Figure 5:**
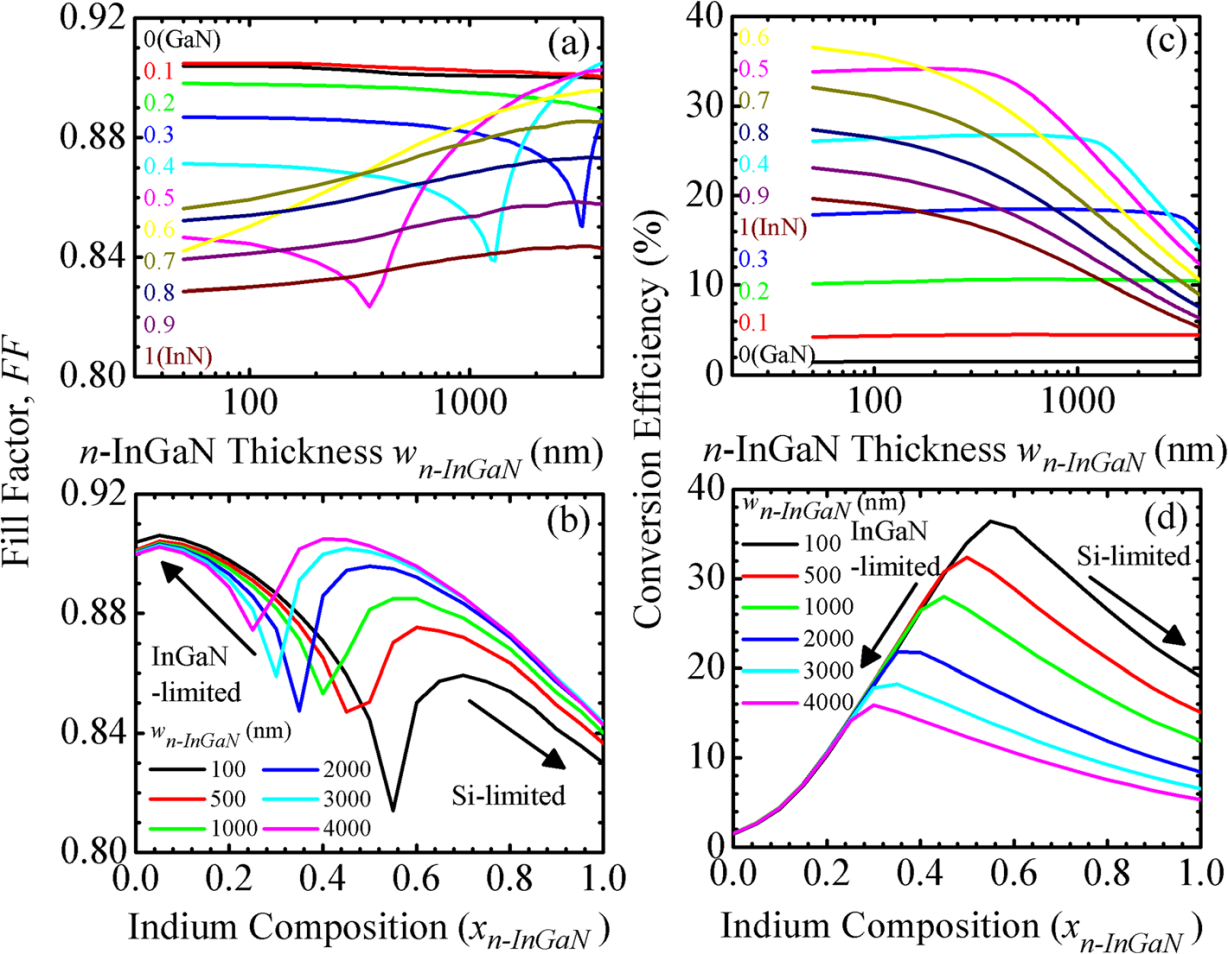
**Fill factor, FF(*w***_***n-*InGaN**_**, *x***_***n-*InGaN**_**).** Of InGaN/Si tandem cells as a function of *n*-InGaN **(a)** thickness, *w*_*n-*InGaN_, and **(b)** indium composition, *x*_*n-*InGaN_. Conversion efficiency, *η*(*w*_*n-*InGaN_, *x*_*n-*InGaN_), as a function of **(c)***w*_*n-*InGaN_ and **(d)***x*_*n-*InGaN_.

Figure 5a,b shows the fill factor, FF(*w*_*n-*InGaN_, *x*_*n-*InGaN_), of InGaN/Si tandem cells as a function of *w*_*n-*InGaN_ and *x*_*n-*InGaN_, respectively. Similar to the previous argument, the FF represents the ratio of *η* to *J*_sc_. In Figure 5a, the FF is divided into low- (*x*_*n-*InGaN_ = 0 to 0.2), medium- (*x*_*n-*InGaN_ = 0.3 to 0.5), and high-indium (*x*_*p-*InGaN_ = 0.6 to 1) InGaN categories:

(I) For *x*_*n-*InGaN_ = 0 to 0.2, as the *w*_*n-*InGaN_ increases, the InGaN subcell-limited FF slightly decreases.

(II) For *x*_*n-*InGaN_ = 0.3 to 0.5, as the *w*_*n-*InGaN_ increases, the FF decreases and then dramatically increases. For $w_{n-\text{InGaN}}<w_{n-\text{InGaN}}^{\text{InGaN}‒to‒Si}$ and $w_{n-\text{InGaN}}>w_{n-\text{InGaN}}^{\text{InGaN}‒to‒Si}$, the FF of InGaN/Si tandem cells are InGaN and Si subcell-limited, respectively. For each composition, minimum FF at the current-matching condition is observed at the $w_{n-\text{InGaN}}^{\text{InGaN}‒to‒Si}$. This $w_{n-\text{InGaN}}^{\text{InGaN}‒to‒Si}$ is the same as $w_{n-\text{InGaN}}^{\text{InGaN}‒to‒Si}$ shown in Figure 4a.

(III) For *x*_*n-*InGaN_ = 0.6 to 1, as the *w*_*n-*InGaN_ increases, the Si subcell-limited FF increases.

In Figure 5b, for $\left(x_{n-\text{InGaN}}\leq x_{n-\text{InGaN}}^{\text{InGaN}‒to‒Si}\right)$ and $\left(x_{n-\text{InGaN}}\geq x_{n-\text{InGaN}}^{\text{InGaN}‒to‒Si}\right)$, the FF of InGaN/Si tandem cells are InGaN and Si subcell-limited, respectively. As the *x*_*n-*InGaN_ increases, minimum FF at the current-matching condition is observed at a certain composition, $x_{n-\text{InGaN}}^{\text{InGaN}‒to‒Si}$. The $x_{n-\text{InGaN}}^{\text{InGaN}‒to‒Si}$ is also determined by the same $x_{n-\text{InGaN}}^{\text{InGaN}‒to‒Si}$, as shown in Figure 4b.

Figure 5c,d shows the conversion efficiency, *η*(*w*_*n-*InGaN_, *x*_*n-*InGaN_), of InGaN/Si *p-n* tandem cells as a function of *w*_*n-*InGaN_ and *x*_*n-*InGaN_, respectively. The *η* is determined by the current-matching effect. In Figure 5c, the trend of *η* is similar to that of *J*_sc_ in Figure 4a. This shows that *J*_sc_ is a dominant factor in determining the performance of InGaN/Si tandem cells. The *η* are divided into low- (*x*_*n-*InGaN_ = 0 to 0.2), medium- (*x*_*n-*InGaN_ = 0.3 to 0.5), and high-indium (*x*_*p-*InGaN_ = 0.6 to 1) InGaN categories:

(I) For *x*_*n-*InGaN_ = 0 to 0.2, the InGaN subcell-limited *η* is slightly higher in the thicker *w*_*n-*InGaN_ tandem cells. This *η* is lower than that of Si solar cell. This shows that the low-indium InGaN/Si tandem cell is not suitable for solar cell applications.

(II) For *x*_*n-*InGaN_ = 0.3 to 0.5, as the *w*_*n-*InGaN_ increases, the *η* slightly increases and then drops at the $w_{n-\text{InGaN}}^{\text{InGaN}‒to‒Si}$. For each composition, maximum *η* at the current-matching condition is observed at the $w_{n-\text{InGaN}}^{\text{InGaN}‒to‒Si}$. With $w_{n-\text{InGaN}}<w_{n-\text{InGaN}}^{\text{InGaN}‒to‒Si}$ and $w_{n-\text{InGaN}}>w_{n-\text{InGaN}}^{\text{InGaN}‒to‒Si}$, the *η* of InGaN/Si tandem cells are InGaN and Si subcell-limited, respectively. With 300 nm *p*-InGaN and 100 to 300 nm *n*-InGaN, the In_0.5_Ga_0.5_N(1.68 eV)/Si(1.12 eV) tandem cell can exhibit as high a *η* as approximately 34%.

(III) For *x*_*n-*InGaN_ = 0.6 to 1, the Si subcell-limited *η* is lower in the thicker *w*_*n-*InGaN_ InGaN/Si tandem cells. With 300 nm *p*-InGaN and 50 nm *n*-InGaN, the In_0.6_Ga_0.4_N(1.42 eV)/Si(1.12 eV) tandem cell can exhibit as high a *η* as approximately 36.5%, which is much higher than the approximately 22% conversion efficiency of an In_0.6_Ga_0.4_N *p*-*n* single junction solar cell and comparable to the 35% to 38% conversion efficiency of a GaInP(1.95 eV)/GaAs(1.42 eV) tandem cell [3,13].

In Figure 5d, as the *x*_*n-*InGaN_ increases, the trends of *J*_sc_, *V*_oc_, and FF lead to the maximum *η* in the medium-In, $x_{n-\text{InGaN}}^{\text{InGaN}‒to‒Si}$, InGaN/Si tandem cells. At $x_{n-\text{InGaN}}^{\text{InGaN}‒to‒Si}$, the *J*_sc_ of the InGaN subcell is equal to that of the Si subcell such that the current-matching condition is obtained for the InGaN/Si tandem cell operating at the maximum power point. Similar to *J*_sc_ and FF, the *η* for low- $\left(x_{n-\text{InGaN}}<x_{n-\text{InGaN}}^{\text{InGaN}‒to‒Si}\right)$ and high-In $\left(x_{n-\text{InGaN}}>x_{n-\text{InGaN}}^{\text{InGaN}‒to‒Si}\right)$ InGaN/Si tandem cells are InGaN and Si subcell-limited, respectively. The In composition corresponding to the optimized *η* is the same as the $x_{n-\text{InGaN}}^{\text{InGaN}‒to‒Si}$ shown in Figure 4b. With 300 nm *p*-InGaN and 100 nm *n*-InGaN, the In_0.55_Ga_0.45_N (1.54 eV)/Si (1.12 eV) tandem cell has the maximum *η* approximately 36.5%. The $x_{n-\text{InGaN}}^{\text{InGaN}‒to‒Si}$ for the maximum *η* is determined by the current-matching effect.

## Conclusions

In summary, we have shown that the performance and characteristics of the InGaN/Si tandem cells are determined by the current-matching effect, which in turn is affected by the *p-* and *n*-layer thicknesses, indium content, and position of depletion region of the InGaN subcell. *J*_sc_ is a dominant factor in determining the performance of InGaN/Si tandem cells. The combined effects of the *J*_sc_, *V*_oc_, and FF lead to an optimized *η* in the medium-indium content, $x_{p\left(n\right)-\text{InGaN}}^{\text{InGaN}‒to‒Si}$, InGaN/Si tandem cell. Similar to *J*_sc_ and FF, the *η* for low- $\left(x_{p\left(n\right)-\text{InGaN}}<x_{p\left(n\right)-\text{InGaN}}^{\text{InGaN}‒to‒Si}\right)$ and high-In $\left(x_{p\left(n\right)-\text{InGaN}}>x_{p\left(n\right)-\text{InGaN}}^{\text{InGaN}‒to‒Si}\right)$ InGaN/Si tandem cells are InGaN- and Si subcell-limited, respectively. With appropriate thicknesses of *p*- and *n*-InGaN, In_0.5–0.6_Ga_0.5–0.4_ N/Si tandem cells can exhibit as high as approximately 34% to 36.5% conversion efficiency. The performance of InGaN/Si tandem cells can be optimized through the optimization of the device structures. Simulation results help us to better understand the current-matching effect and operation mechanisms of InGaN/Si tandem cells.

## Competing interests

The authors declare that they have no competing interests.

## Authors’ contributions

CML prepared the theoretical formula and did the theoretical simulation. CYT and LWT made result discussions. SWF coordinated the project and drafted the paper. All the authors read and agree the final version of the paper.
